# Dietary Patterns, Skeletal Muscle Health, and Sarcopenia in Older Adults

**DOI:** 10.3390/nu11040745

**Published:** 2019-03-30

**Authors:** Antoneta Granic, Avan A. Sayer, Sian M. Robinson

**Affiliations:** 1 AGE Research Group, Institute of Neuroscience, The Medical School, Newcastle University, Newcastle upon Tyne NE2 4HH, UK; antoneta.granic@newcastle.ac.uk (A.G.); avan.sayer@newcastle.ac.uk (A.A.S.); 2 NIHR Newcastle Biomedical Research Centre, Newcastle upon Tyne Hospitals NHS Foundation Trust and Newcastle University, Campus for Ageing and Vitality, Newcastle upon Tyne NE4 5PL, UK; 3 Institute for Ageing, Newcastle University, Newcastle upon Tyne NE2 4AX, UK

**Keywords:** dietary patterns, nutrition, ‘myoprotective’ diet, Mediterranean diet, sarcopenia, muscle strength, muscle mass, muscle function, older adults

## Abstract

In recent decades, the significance of diet and dietary patterns (DPs) for skeletal muscle health has been gaining attention in ageing and nutritional research. Sarcopenia, a muscle disease characterised by low muscle strength, mass, and function is associated with an increased risk of functional decline, frailty, hospitalization, and death. The prevalence of sarcopenia increases with age and leads to high personal, social, and economic costs. Finding adequate nutritional measures to maintain muscle health, preserve function, and independence for the growing population of older adults would have important scientific and societal implications. Two main approaches have been employed to study the role of diet/DPs as a modifiable lifestyle factor in sarcopenia. An a priori or hypothesis-driven approach examines the adherence to pre-defined dietary indices such as the Mediterranean diet (MED) and Healthy Eating Index (HEI)—measures of diet quality—in relation to muscle health outcomes. A posteriori or data-driven approaches have used statistical tools—dimension reduction methods or clustering—to study DP-muscle health relationships. Both approaches recognise the importance of the whole diet and potential cumulative, synergistic, and antagonistic effects of foods and nutrients on ageing muscle. In this review, we have aimed to (i) summarise nutritional epidemiology evidence from four recent systematic reviews with updates from new primary studies about the role of DPs in muscle health, sarcopenia, and its components; (ii) hypothesise about the potential mechanisms of ‘myoprotective’ diets, with the MED as an example, and (iii) discuss the challenges facing nutritional epidemiology to produce the higher level evidence needed to understand the relationships between whole diets and healthy muscle ageing.

## 1. Introduction

The world’s population is getting older [1]. Population ageing has been regarded not only as one of the greatest accomplishments of humankind but also as a societal challenge to establish how to increase health span and create the potential for the added years of life to be characterised by good health and functioning [2]. Based on the 2017 report from the United Nations [1], the number of older adults aged 60 and over (≥60) will increase worldwide from 962 million (or 1 in 8 people) in 2017 to 2.1 billion (1 in 5) by the middle of the century. Advanced age is the main risk factor associated with the development of chronic diseases such as cardiovascular, respiratory, neurological, and musculoskeletal diseases. However, there is great heterogeneity in ageing and health in later life [3,4,5], which indicates an opportunity for preventive strategies to increase healthspans [3]. Several environmental and lifestyle factors have been recognised to modify the ageing process [6], including physical activity [7,8] and diet [9,10,11,12,13,14,15,16]. 

The major threats to achieving a healthy lifespan are age-related physiological impairments in multiple organs, which lead to functional limitations, disability, and reduced quality of life, as well as exacerbating the risk and severity of age-related chronic diseases [10]. Alterations in body composition characterised by a decrease in skeletal muscle mass and increase in body fat are among the most profound age-related changes [17,18]. These start in mid-adulthood and progress gradually thereafter. An estimated annual loss of muscle mass of 3% after the age of 60 is accompanied by a preferential loss of fast type II myofibres and their conversion into slow type I fibres, along with denervation of motor units and fat infiltration between and within the fibres [17,18]. Though the losses of muscle strength and power with ageing are even greater [19,20], longitudinal studies have shown a wide inter-individual variability in both muscle mass and strength decline in older adults [21,22]. This highlights the potential importance of modifiable risk factors such as physical activity, diet, and nutrition as influences on muscle ageing [23,24,25]. 

Sarcopenia, a muscle disease characterised by low muscle strength, mass, and quality [26,27] is associated with functional decline, poor quality of life, and increased mortality [28,29,30,31,32]. Sarcopenia has a complex aetiology that is not fully elucidated. Multifactorial environmental and genetic factors, including low grade chronic inflammation [33,34], insulin [35,36] and anabolic resistance [37,38,39], hormonal changes [40,41], mitochondrial dysfunction [36,42], oxidative stress [42,43], malnutrition [24,44,45,46], inactivity [47,48], and age-related chronic diseases [49,50,51] contribute to progressive and adverse changes in ageing muscle. Intervention studies suggest that some of these pathophysiological processes may be counteracted by ensuring adequate nutrition and exercise levels [38,42,47,48]. 

An extensive number of observational and intervention studies have used a single nutrient approach to investigate the relationship between diet and muscle health with ageing (e.g., protein or vitamin D with or without exercise; [44,52,53,54]). However, separating the influence of one dietary component from others and understanding their interactions within a whole diet on health outcomes is challenging. Only recently, studies using a whole diet approach have emerged to understand the role of diet/dietary patterns (DPs) in muscle health with ageing [55,56,57,58,59]. This approach acknowledges the complexity of human consumption in relation to health [60], and the complexity of understanding the effects of nutrient and bioactive components within a whole diet [61,62,63] when examining their influence on ageing muscle. 

To date, two main methods have been used to explore the DP-muscle health link in epidemiological studies of muscle ageing: A priori (hypothesis-driven) and a posteriori (data driven) methods to identify DPs. DPs derived a priori are defined by a higher adherence to predefined dietary scores or indices (e.g., the Mediterranean-style diet (MED) [15,16,64], indices of diet quality (e.g., Healthy Eating Indices) [56]) that are based on current knowledge about what constitutes a healthy diet for, for example, cardiovascular and general health. Higher scores reflect a diet characterised by higher consumption of beneficial foods (e.g., fruits and vegetables, whole grains, lean meat, fish, nuts, low-fat dairy, and olive oil) and their combination and low consumption of nutrient-poor foods (e.g., refined grains, sweets, processed meats, and sources of trans fats) [65,66,67]. Conversely, the a posteriori method is purely exploratory; it uses all available dietary data to derive DPs and may therefore better define the usual diet in a population, which may or may not relate to health outcomes. The a posteriori method utilises multivariate statistical tools (i.e., data reduction techniques), such as factor/principal component (PCA) and cluster analysis to explore DPs. These techniques are markedly different procedures but can be used in a complementary way to improve the interpretability of the results obtained from each method [68]. Reduced Rank Regression (RRR)—a newer hypothesis-driven approach, not yet used in nutritional epidemiology of muscle ageing [63,69]—identifies foods/food groups in a population that best explain the variation in a priori selected variables hypothesised to be independently associated with disease aetiology, such as nutrient intakes (e.g., protein or vitamin D for muscle health) or biomarkers implied in disease pathogenesis (e.g., inflammatory cytokines). 

Epidemiological investigation into DPs can contribute to a knowledge base for the development of interventions for optimal muscle ageing [56,57,58,59], and may inform nutritional public policy aimed to promote healthy ageing. Therefore, the purpose of this review is to (i) summarise current evidence in nutritional epidemiology from four recent systematic reviews (cut-off for inclusion April 2017) about the role of DPs in muscle health, sarcopenia, and its components, and to update this evidence with new primary studies published since then; (ii) hypothesise about the potential mechanisms of a ‘myoprotective’ diet, with a focus on the MED as an example; and (iii) discuss the challenges facing nutritional epidemiology to produce the higher level evidence needed to understand relationships between whole diets and healthy muscle ageing.

## 2. Evidence about the Role of Dietary Patterns in Muscle Ageing Studies 

To our knowledge, there are four systematic reviews (publication years 2017–2018; cut-off for inclusion April 2017) that have examined evidence from nutritional epidemiology about the role of DPs in muscle health and function in older adults [56,57,58,59]. Only one examined the relationship between diet quality (defined both a priori and a posteriori) and components of sarcopenia; this included studies that used various Healthy Eating Indices (e.g., HEI, Canadian HEI (C-HEI), Alternative HEI (AHEI), HEI-2005, Healthy Diet Indicator (HDI), Dietary Variety Score (DVS), and Diet Quality Index-International (DQI-I)) along with region-specific DPs such as the MED and Nordic diet (Nordic Diet Score, NDS) [56]. The remaining reviews [57,58,59] summarised the evidence from studies investigating the associations between the MED and muscle health outcomes across the life course [57] and in older adults [58,59]—the MED being a ‘healthy’ DP most frequently used in observational studies of muscle ageing and showing most consistent evidence, and thus hypothesised here to be ‘myoprotective’. 

### 2.1. Dietary Patterns Derived A Priori: Example of Mediterranean Diet

#### 2.1.1. Main Characteristics of Mediterranean Diet

Of all healthy DPs determined a priori, the MED has been most recognised because of its beneficial effects on various health outcomes including survival, cardiovascular disease, cognitive health, frailty, and cancer [15,16,64,70,71,72,73,74,75]. A DP traditionally associated with populations living around the Mediterranean Sea in olive grove regions, the MED is characterised by a higher consumption of plant foods (fruits, vegetables, legumes, and cereals) and olive oil, a moderate intake of fish, eggs, poultry, and dairy foods, a low intake of red meats, and a moderate consumption of red wine during meals [76]. Declared by the United Nations Educational, Scientific and Cultural Organization (UNESCO) (2010) [77] to be an Intangible Cultural Heritage of Humanity, the MED as a lifestyle includes not only beneficial foods for health but also intense physical work/exercise and tight social links through sharing of skills, knowledge, and traditions about, among others, crops, harvesting, fishing, food preservation, cooking, and food consumption [78]. Though the adherence to MED-style DPs has been assessed using different variants of MED indices (scores) [79,80] in various populations (including those living outside the Mediterranean basin), scientific consensus exists about the fundamental components of the MED today [81]. The main principles of the MED rest on its plant-based core, moderation in food consumption, and cultural and lifestyle elements such as cooking, conviviality, physical activity, and adequate rest [81]. The benefits of the MED for general health and quality-of-life have been recently been recognized in national dietary guidelines such as the 2015–2020 Dietary Guidelines for Americans [82]. 

#### 2.1.2. Systematic Reviews of Mediterranean Diet and Muscle Ageing: Summary of Evidence

In the last decade, a number of population-based cross-sectional and longitudinal studies have investigated the role of the MED in skeletal muscle health (i.e., mass, strength, function, physical performance, and sarcopenia) in older adults. Along with general, cardiovascular, metabolic, and cognitive health benefits, MED-style DPs may offer ‘myoprotective’ properties. Four recent systematic reviews (publication years 2017–2018) have synthesised the best available evidence exploring MED-muscle ageing links [56,57,58,59]. These reviews identified largely consistent positive associations with several muscle-related outcomes, especially with the measures of muscle functioning (Table 1), with these effects seen both in Mediterranean and non-Mediterranean populations. However, some challenges in understanding the MED pattern have been noted, including (i) a great variation in the MED indices used to define DP (exposure) in relation to different muscle outcomes across populations; (ii) variability in both the dietary and muscle-related assessments used to measure exposures and outcomes, respectively; (iii) differences between studies in the covariate factors employed to account for confounding; (iv) paucity of longitudinal studies; and (v) a small number of studies using sarcopenia as an outcome compared with other age-related conditions [24]. Taken together, these limitations have contributed to the lack of more precise quantitative reviews of the effect of the MED on sarcopenia and its components in older adults, particularly opportunities for using pooled data. 

In a systematic review that investigated the relationship between the MED and musculoskeletal health over the life course [57], 18 studies were selected; however, only two explored the association between the MED score (MDS)—originally described by Trichopoulou et al. [70]—and muscle-related health in older adults [83,84]. The first of these was a prospective cohort study of over 2900 older men and women aged ≥65 living in Hong Kong [83]. In this study, a one-unit increase in MDS was not associated with prevalent or four-year incident sarcopenia (defined by the Asian Working group for Sarcopenia algorithm [106]) after adjustment for socio-demographic, health, and lifestyle variables. Despite some similarities between the Chinese diet and the MED (e.g., a higher intake of fruits and vegetables and a low intake of meats), the authors attributed the lack of association to a very low consumption of food groups traditional for the MED in this cohort (e.g., legumes, nuts, olive oil, and wine). Because the MDS is a sum of nine or ten binary components representing food groups deemed to be either beneficial (scoring 1) or detrimental (scoring 0) for overall health based on sex-specific median intakes, the cohort-specific medians in this study may not have represented healthy levels of intake. In contrast, the study of over 2500 women aged 18–79 from the TwinUK registry found that the percentage of fat-free mass (FFM/weight × 100) in women aged ≥50 was positively associated with the highest MDS quartile compared with the lowest quartile, with a 13% difference in leg extension power between the extreme quartiles after adjustment for key covariates [84]. 

A review by McCure and Villani (2017) [59] synthesised the evidence from an additional eight studies, although much of this was cross-sectional (five cross-sectional, two longitudinal, and one cross-sectional and longitudinal), in older adults aged ≥55 [85,86,87,88,89,90,91,92]. The MED was assessed using MDS (four studies) [85,86,87,88,89] and other MED score variants (e.g., Alternate Mediterranean Food Score, Mediterranean-type Diet Score) [87,89,90,91,92]. The muscle-related outcomes varied across the studies, from walking speed, lower body physical performance (Short Physical Performance Battery (SPPB)), skeletal muscle mass, quality, and strength (grip strength) [86,87,88,89,90,91,92], and by several indices of sarcopenia [85]. The studies included older adults living in Mediterranean (e.g., Italy, Mediterranean islands) and non-Mediterranean regions (e.g., United States, Germany, Finland). The length of follow-up in the longitudinal studies was 3–9 years. All studies, both cross-sectional and longitudinal, reported benefits of adherence to the MED, such that there was a lower risk of sarcopenic symptomology with greater adherence to the MED [85,86,87,88,89,90,91,92]. Higher MED scores were associated with better muscle health and function [85,88,89,90,91,92], and reduced risk of decline in lower extremity functioning [86] and mobility [87]. 

For example, the study of over 550 women aged 65–72 living in Kuopio, Finland, found that those in higher quartiles of MDS had faster walking speed and greater lower body muscle quality (10 m-walking speed per leg lean mass) at baseline. Women in the lowest MDS quartile had a greater decline in relative skeletal muscle index (RSMI) and total lean mass (LM) over a 3-year follow-up compared with those in higher quartiles [85]. In the study of over 930 Italian men and women (Invecchiare in Chianti, InCHIANTI Study) aged ≥65 years, higher adherence to the MED was associated with less decline in lower extremity performance (SPPB) over 3, 6, and 9-year follow-ups [86]. Those with the highest MDS (score 6–9) had about one point higher SPPB score at each follow-up compared to those with the lowest MDS (score ≤3) after adjustment for a set of covariates. Importantly, a one-point decline in SPPB represents a clinically relevant change in lower body function. Additionally, participants with higher MED adherence had a 29% lower risk of developing mobility disability (a SPPB score of <10 points on the scale of 0–12) over the study period [86]. In the Health, Aging, and Body Composition Study (Health ABC) of over 2200 participants aged 70–79, both usual and rapid 20 m walking speed were higher in those with greater MED adherence (MDS 6–9). A higher MDS was associated with less decline in usual walking speed over 8 years after adjustment for key covariates, although this was attenuated by adjustment for total body fat percentage [87]. In contrast, the association was not changed for MED adherence and rapid 20 m walking speed over the study period. Both of these longitudinal studies [86,87] used the same statistical approach and adjusted for a similar set of confounders and have estimated MED exposure (MDS score) from food frequency questionnaires (FFQ); importantly they have also investigated the MED and muscle function relationship in Mediterranean [86] and non-Mediterranean populations [87]. Their findings provide strong support for the MED; greater adherence to the MED may help with mobility and slow the decline in lower body function in older adults. 

The third systematic review and meta-analysis that investigated the relationship between the MED, frailty, functional disability, and sarcopenia in older adults [58] included two additional prospective cohort studies [93,94] (publication year 2018). In the Senior-ENRICA study of over 1600 older adults aged ≥60 years, higher adherence to the MED assessed by MDS was not associated with the risk of mobility impairment and agility (defined using the Rosow and Breslau scale) or physical functioning decline (a ≥5-point decline on the physical component of the 12-Item Short-Form Health Survey (SF-12)) over three-year follow-up [93]. However, when accordance to the MED was assessed using the Mediterranean Diet Adherence Screener (MEDAS)—a 14-component score used in the PREDIMED (PREvención con DIeta MEDiterránea) trial [64]—the highest MEDAS tertile was associated with 23–40% decreased odds for developing impairments in agility and mobility as well as a decrease in physical functioning [93]. In the Three-City-Bordeaux study of 560 initially non-frail older adults aged ≥65 years, the highest MDS (score 6–9) was associated with a 55% reduced risk of developing mobility impairment (defined using the Rosow and Breslau scale) and a 56% lower risk of lower extremity impairment (chair stands) over a two-year follow-up [94].

The fourth systematic review included evidence from observational studies investigating the relationship between DPs defined a priori and a posteriori (including the MED) and sarcopenia/elements of sarcopenia [56]. Two additional studies from InCHIANTI [73] and Senior-ENRICA cohorts [1] using MDS and MEDAS scores were included in this review and reported similar findings as in Milaneschi et al. [86] and Struijk et al. [93]. A higher adherence to the MED (MDS ≥ 6) was associated with a 51% lower risk of low walking speed compared with low adherence (MDS ≤ 3) after a six-year follow-up. There was no association between muscle strength (grip strength) and MDS [73]. Similarly, no significant association between MDS, grip strength, and walking speed was observed in the ENRICA cohort, but belonging to the highest MEDAS tertile was associated with a 47% reduced risk of low walking speed [95].

Taken together, the reviews provide strong evidence for the beneficial effects of the MED, with better physical functioning, including lower body function (e.g., SPPB) and mobility (walking) observed across the studies, suggesting that higher adherence to an MED-like DP may be ‘myoprotective’ in older adults. Differences in findings between some studies (e.g., [93]) may illustrate the complexity in deriving a Mediterranean-type DP and the lack of a uniform operational definition for the MED score. Debate continues about which components of the MED should be included—which type (e.g., fat and grain), amounts and cut-offs for intake of food groups (e.g., alcohol) should be used in the score for the Mediterranean versus non-Mediterranean populations [107,108]. The current diet of the Mediterranean regions differs from the traditional MED originally described in Greece in 1960s [70]. To understand and compare the influence that higher adherence to the MED may have on skeletal muscle health in older adults, a univocal definition of the MED and its dietary score based on modern understanding of the MED should be established [81], one of which may possibly include non-traditional MED foods. 

#### 2.1.3. Mediterranean Diet and Muscle Ageing: Additional Evidence

To update the findings for the associations between the MED and sarcopenia/components of sarcopenia, we conducted additional literature searches of peer-reviewed, full-length articles published in English since April 2017 (electronic databases searches of PubMED, MEDLINE, SCOPUS, and OVID; and cut-off for inclusion January 2019). We used the search strategy and key words similar to those described by McCure et al. [59], and included observational studies (cross-sectional and prospective) in older adults aged ≥60 years that investigated the MED and at least one muscle health outcome related to sarcopenia. We identified four more recent primary studies that investigated the role of the MED in muscle health and function in older adults [99,100,101,102] (Table 1). In a study of 380 Spanish older adults aged 50–80, the highest quartile of the MED (derived a posteriori by factor analysis) was positively associated with 30-s chair stands in men and a six-min walking speed in women but not with the measures of muscle strength (grip strength and arm curls) in both sexes. Additionally, men in the highest MED quartile needed less time to complete the eight-foot Timed Up-and-Go Test (TUG) and had faster 30-m gait speed compared to those in lower quartiles, whilst those belonging to higher quartiles of ‘Westernized’ DP were associated with slower gait speed, lower body strength (chair rises), aerobic endurance (six-min walk test) and agility (eight-foot TUG) in men and women [99]. Conversely, a cross-sectional study of 84 Italian older women found that those with higher grip strength (>20 kg) had significantly higher PREDIMED scores determined from the seven-day food records and were more likely to belong to the ‘high adherence’ PREDIMED group (score ≥10) [100]. In contrast, in the Helsinki Birth Cohort prospective study of over 960 community-dwelling older adults (mean age 61.6 years), higher adherence to modified MDS at baseline was not associated with mobility limitations at 10-year follow-up [101]. In addition, although a cross-sectional study of 117 type-2 diabetes patients aged >60, recruited through the Center for Successful Aging with Diabetes at the Sheba Medical Center in Israel, found that those with higher adherence to MDS (score 5–9) had higher grip strength and reduced risk of falls; the associations did not persist in multi-variable analyses that adjusted for key covariates. However, there was a significant age by MDS interaction in this study, such that being in the highest tertile of MDS was associated with longer distance achieved in six-minute walking test, faster time needed to a complete 10-m walk, and a better balance score, but this was only found in patients aged ≥75 years [102] (Table 1).

In summary, the observational evidence from the additional primary studies reviewed, for the benefits of MED-type DPs for muscle health in older adults, is largely consistent with the findings of the systematic reviews. Whilst there are few studies that have investigated the role of the MED (using MED scores) in the aetiology of sarcopenia [83,85], a number have explored the relationship between components of sarcopenia (e.g., grip strength, walking speed, and muscle mass) and a decline in physical function (e.g., [86,87,90,91,93,100,101,102]). Higher adherence to the MED (assessed by MDS, PREDIMED score, and other variants) was positively associated with lower extremity functioning, mobility, and better walking speed over time [84,86,87,89,91,93,94,99,102] but not with measures of upper body muscle strength in most of the studies [73,91,95,99,102]. These results suggest that whilst the MED may not improve muscle strength in older adults, higher adherence to an MED-like DP may be beneficial for mobility and general physical functioning. However, as positive associations between muscle strength and the MED have been observed in some studies (e.g., [100] in older women aged 60–85 using PREDIMED score) these need to be further explored to be replicated in other cohorts. Despite the gaps in current knowledge, the MED may have the greatest potential to be ‘myoprotective’, although to reach a higher-level of evidence, internal and external validations of MED indices across prospective studies with longer follow-ups taking a life course approach are needed to support future clinical trials of ‘myoprotective’ DPs in older adults. 

### 2.2. Dietary Patterns Derived A Priori: Example of Diet Quality Indices

The systematic review by Bloom et al. (2018) [56] included eight studies that used 10 different indices of diet quality other than the MED (e.g., HEI, C-HEI, AHEI, HEI-2005, DVS, HDI, DQI-I, and NDS) in relation to sarcopenia/elements of sarcopenia in older adults (Table 1). Diet was assessed mostly by FFQ (five studies), followed by 24-h recall and three-day weighed food records. Muscle health-related outcomes varied from appendicular skeletal muscle mass (ASM), muscle strength (e.g., grip strength, knee extensor), physical performance (e.g., gait speed, senior fitness test, SPPB) to sarcopenia. Seven of the studies used dietary indices that are based on scientific evidence about DPs for general health and compliance with population dietary guidelines for recommended reference intakes of nutrients (e.g., HEI and HEI-2005 recommended by the Diary Guidelines for Americans and the US Department of Agriculture [109]). Specifically, HEI (and the updated versions) has used a universal scoring metric and density approach to calculate amounts of a set of foods per 1000 kcal to assess the quality of diet in the US population. HEI includes food groups that are culturally neutral; scores are given for total fruits and vegetables, whole and refined grains, total protein foods, seafood and plant-based protein foods, calories from solid fats, added sugar, sodium, and alcohol [109]. The HEI is widely used in different research settings and in relation to an array of outcomes, including muscle health in older adults. 

For example, in the National Health and Nutrition Examination Survey (NHANES 1999–2002) of over 2000 older adults aged ≥60 years, diets with scores in the higher quartiles of HEI-2005 were associated with higher knee extension power and faster gait speed compared with the lowest HEI quartile. However, the associations were no longer significant after adjustment for physical activity [96]. In a study of community-dwelling older Australians (aged ≥60 years) lean mass and physical performance (SPPB) were not associated with HEI-total score, but, in contrast, a weak positive association between lean mass in women and SPBB in men was observed when HDI was used to assess diet quality [97]. In a study of over 150 older adults (mean age at baseline 74.6 years) with type 2 diabetes, a higher adherence to C-HEI was not associated with maintenance of muscle strength (grip strength, knee extensor and elbow flexor strength) over three years [98]. To date, only one study has examined the association between diet quality index (DQI-I) and the risk of sarcopenia. In a cohort of community-dwelling Chinese older adults aged ≥65, men in the highest quartile of DQI-I had a 50% reduced risk of prevalent but not four-year incident sarcopenia compared with those in the lowest quartile. No significant association between DQI-I and sarcopenia was observed in women [83]. 

#### 2.2.1. Diet Quality Indices and Muscle Ageing: Additional Evidence 

Using the literature search strategy described previously (Section 2.1.3), we identified four additional primary studies (three longitudinal and one cross-sectional) that used diet quality indices or region-specific diet scores in relation to muscle strength and function [101,103,104,105] in older adults aged ≥60 years at baseline (Table 1). In the Helsinki Birth Cohort study, the likelihood of developing mobility limitations was 58% lower in older adults in the highest tertile of NDS compared with those in the lowest tertile when assessed 10 years later [101]. The NDS included food groups that are more reflective of the diets consumed in the Nordic countries, such as berries, root and cruciferous vegetables, rye bread, and a high intake of fish. In the same cohort, each one-unit increase in NDS was associated with a 1.44 Newton (N) higher grip strength and a 1.83 N higher leg strength in women, but not in men, at a 10-year follow-up [103]. No associations were found with muscle mass in either sex [103]. In the British Regional Heart Study of over 1200 men (mean age 66 at baseline), being in the highest category versus the lowest category of HDI (score 4–8 versus score 0–1) and Elderly Dietary Index (EDI; score 27–36 versus score 9–22) was associated with a 45% and a 50% reduced risk of mobility limitations, respectively, 15 years later [104]. In a cross-sectional study of over 600 Korean older adults (aged ≥65 years) participating in the National Fitness Award project, higher Recommended Food Score (RFS) was positively associated with grip strength in women but not in men. No associations were observed between the RFS and other physical performance tests, including two-min step test, TUG, figure-of-eight walk test, and arm curls. The study used a modified RFS to include foods characteristic of Korean diet, such as seaweed [105]. 

In summary, the findings of the studies that used indices of diet quality in relation to components of sarcopenia included in the systematic reviews have been mixed; however, the positive findings for mobility/mobility limitations from the recent prospective studies need further exploration [101,103,104]. Most of the indices to date are based on a combination of foods and nutrients intake recommended to promote general health. The concept of ‘diet quality’ is multidimensional and heterogeneous, and there is a great variety across the scores regarding food groups and amounts [110]. There is a possibility that a ‘diet quality’ concept and what may constitute a healthy diet to protect other outcomes, such as cardiovascular health, may not be optimal for healthy muscle ageing. To test this hypothesis, additional evidence from prospective cohort studies with longer follow-up are needed, together with validation and greater harmonisation of diet quality indices across the populations. 

### 2.3. Dietary Patterns Derived A Posteriori 

The systematic review by Bloom et al. (2018) [56] provided the most comprehensive synthesis of evidence from observational studies that used an a posteriori (data driven) approach to derive DPs and to explore their relationship with sarcopenia [111] and its components of sarcopenia [112,113,114,115,116,117] (Table 2). Four cross-sectional [111,112,113,115] and three longitudinal studies [114,116,117] were included (duration of follow-up between 3.5 and 16 years) that used data reduction techniques (i.e., PCA/factor analysis [111,113,114,115,117], and clustering [112,116]) to derive DPs from dietary data assessed either by FFQ [111,113,114,115], 24-h diet recall [112,116], or diet history [117]. Similar to the studies using MED scores for dietary exposures, muscle health-related outcomes varied across the studies from muscle mass [112], strength (grip strength) [113,116,117], physical performance (walking speed, chair rises, one-leg balance, TUG) [114,115,116,117], and sarcopenia [111]. Healthier DPs were generally described by higher intake of beneficial foods for overall health (e.g., higher intake of fruits and vegetables, fatty fish, and whole grains) in studies of Korean, British, and Spanish older adults. 

DP, dietary pattern; FFQ, food frequency questionnaire; MRC, Medical Research Centre; PCA, principal component analysis; KNHANES, Korea National Health and Nutrition Examination Survey; TUG, Timed Up-and-Go Test.

Differences in how dietary exposures and muscle health outcomes were defined across the studies restricted a direct comparison of results. The review found limited evidence of a relationship between lean body mass and healthier DPs. However, in a study of over 1400 Korean older adults aged ≥65 years, a ‘Westernized Korean’ DP characterised by a higher intake of bread, eggs, fish, milk, grains other than rice, and alcohol was associated with a 74% increased low ASM (i.e., below ASM/weight reference value of 20–39; measured by DXA) compared with a ‘Traditional Korean’ DP high in white rice but low in protein, fat, and milk products [112]. 

Inconsistent evidence has been found for associations between muscle strength (grip strength) and a healthier DP [95,113,116]. A ‘Prudent’ DP, similar to a ‘healthier’ pattern defined by HEI, was associated with higher grip strength in older men and women participating in the Hertfordshire Cohort Study (HCS), which was partially explained by greater intake of fatty fish in men but not in women [113]. Conversely, a ‘Prudent diet’ characterised by high intake of vegetables, potatoes, blue fish, meat, pasta, and olive oil was not associated with grip strength in the ENRICA cohort of Spanish older adults compared to ‘Westernized’ pattern high in refined bread, red and processed meats, and whole dairy products [117]. These findings were similar to the results reported in Struijk et al. [93] using the MED score (MDS) in this cohort, showing the value of using a complementary DP methodology in the study of diet in relation to muscle health. In a cohort of very old adults (aged ≥85), The Newcastle 85+ Study, men belonging to a DP high in red meat, potatoes, and gravy (‘High Red Meat’) had lower grip strength and greater grip strength decline over a five-year follow-up compared to those in a ‘Low Meat’ DP, a diet characterised by a low intake of these foods but a higher intake of fruits, fish/seafood, whole grains/cereal products, dairy foods, and soups [116]. 

More consistent evidence has been reported for a relationship between a less healthy DP and an increased risk of decline in physical performance. For example, being in the highest tertile of a ‘Westernized pattern’ was associated with an 85% increased risk of slow walking speed in the ENRICA cohort [95]. This is consistent with a UK study of over 5300 men aged ≥60, in which being in the highest tertile of a ‘Western-type’ dietary pattern was associated with a 45% increased odds of slow walking speed compared with the lowest tertile, whilst no association between eight-feet walking speed and a ‘Healthy-food’ DP was observed [114]. Similarly, in the Newcastle 85+ Study, women belonging to a DP high in butter (‘High Butter’) had worse TUG performance compared to those in a ‘Low Meat’ DP [118]. 

We are aware of only one cross-sectional [111] and one prospective study that investigated the association between DP derived a posteriori and sarcopenia [118]. In a cross-sectional study of 300 Iranian older adults aged ≥55 years, those in the highest tertile of an MED-style DP identified using PCA (with high factor loadings for MED foods such as olives/olive oil, fruits, vegetables, nuts, whole grains, and fish) had a 58% reduced risk of prevalent sarcopenia. In this study, higher compared with lower adherence to the Western DP (with high factor loadings for tea, sugar, desserts and sweets, soy, and fast food) was not associated with sarcopenia [111]. In the community-dwelling participants from the Newcastle 85+ Study, belonging to a DP characterised by elements of a traditional British diet (high intake of butter, red meats, gravy, potato, vegetables, and sweets/desserts) was associated with a 2.4-fold increased risk of sarcopenia at three-year follow-up compared with a DP in which more people ate unsaturated spreads and oils. A Traditional British DP was also associated with a 2.1-fold and a 5.4-fold increased risk of prevalent sarcopenia at baseline and three-year follow-up, respectively, even in participants with adequate protein intakes [118]. 

#### 2.3.1. Dietary Patterns Derived A Posteriori and Muscle Ageing: Additional Evidence 

We identified four additional primary studies (two longitudinal and two cross-sectional) that explored the relationship between DPs derived a posteriori (data-driven) and muscle health (cut-off for inclusion January 2019) [119,120,121,122] (Table 2). In the prospective Three-City Bordeaux Study of community-dwelling older adults aged ≥67 years, belonging to a ‘Biscuits and snacking’ cluster was associated with a three-fold increase risk of mobility limitations (Rosow-Breslau scale) compared with a ‘Healthy’ cluster (characterised by higher intake of fish in men and fruits in women) over 10 years [119]. Similarly, older Korean men (aged ≥60 years) belonging to a ‘Healthy’ DP (characterised by higher intake of fruits, vegetables, seaweed, mushrooms, legumes, whole grains, potatoes, fish, dairy products, eggs, and red meat) had higher muscle mass compared to those belonging to a ‘Westernized’ DP (high in fast food, rice cake, bread, noodles, rice, red meat, poultry, and soft drinks) [120]. In a cross-sectional study of over 500 Spanish older adults aged ≥70 years, three dietary clusters with a progressively worse adherence to dietary recommendations were identified using multiple correspondence and cluster analysis. A gradient effect of poor physical performance (TUG) was observed from cluster one to cluster three (poorest diet) [121]. This is also consistent with the findings from a cohort of British adults born in 1946 from the MRC National Survey of Health and Development (NSHD) study, in which diet quality identified by PCA at age 36, 43, 53, and 60–64 was associated with better physical performance at age 60–64 years. In particular, higher diet quality scores characterised by higher intakes of fruit, vegetables, and wholegrain bread at age 60–64 were associated with faster chair rise speed and better balance [122]. 

Though emerging, the evidence from studies employing an a posteriori approach to derive DPs in relation to muscle ageing has been mixed (Table 2). The strongest evidence has been found between westernized DPs and impairments in mobility and physical performance. The westernized diets may have increased the risk of poor physical functioning via potential effects on myofibre quality and composition [17,18] and increasing the pathophysiological processes implicated in sarcopenia and functional decline, including fat infiltration [123,124], inflammation [33,34,125,126,127], oxidative stress [127,128], and insulin resistance [129,130,131]. 

To characterise ‘myoprotective’ diets in older adults, a harmonisation of the findings across well-designed prospective cohort studies with longer follow-up and a life course approach [122] that use complementary DP methodology to investigate the diet-muscle health relationships will be essential to enable meaningful pooled analyses currently lacking for sarcopenia and its components [58]. Of all DPs that are either hypothesis or data-driven, only one meta-analysis has been conducted with the MED and functional disability (defined by SPPB, the Rosow and Breslow scale, and SF-12 scores) and frailty as outcomes [58]. Though showing most consistent evidence for components of sarcopenia related to muscle function, studies investigating the MED and sarcopenia were scarce and had different methodological design, thus limiting meaningful comparison [58]. 

### 2.4. Similarities and Differences between the Findings of Dietary Patterns and Muscle Ageing Studies 

Direct comparisons of the associations (positive and negative) between DPs (derived a priori or a posteriori) and muscle health/function in observational studies summarised in the latest reviews [56,57,58,59] and presented here are limited for several reasons. The studies varied by the (i) dietary assessments used to measure the exposure (from FFQs, 24-h recalls to weighed food records); (ii) methods employed to derive DPs (MED indices being the commonest approach, followed by diet quality indices); (iii) assessments used to measure the outcomes (sarcopenia and its components); (iv) muscle health/function outcomes (from body composition and muscle mass (ASM), muscle strength (grip strength, knee and elbow extensor strength), physical performance (gait speed, SPPB, senior fitness test, mobility impairment and agility, physical components of SF-12)); (v) covariates selected to adjust for confounding; (vi) populations under study, including differences in age, background, diet, and nutritional status; and (vii) study designs (more cross-sectional than longitudinal). To our knowledge, the role of DPs in sarcopenia prevalence and incidence has been investigated only in the following four studies [83,85,111,118] in Chinese [83], Nordic [85], Iranian [111], and British [118] older adults, warranting further investigations. A growing literature on DPs recognises the importance of a whole food approach to understand the synergistic, antagonistic, and cumulative effects of nutrients and other food components in muscle health with ageing. However, how the complex concept of a ‘healthy diet for muscle health’ has been addressed varies significantly across studies, from the MED and various HEIs (hypothesis driven and based on general health) to data-driven approaches that describe whole diets in a population. Most studies have used several muscle-related outcomes, but only a few employed different methods within studies to derive DPs to provide complementary findings (e.g., [83,95,97]). 

The most consistent results were observed with MED-type DPs (derived a priori or a posteriori) in relation to physical functioning (lower extremity functioning, mobility, walking speed) in the populations living in the Mediterranean and non-Mediterranean regions [84,86,87,89,91,93,94,99,120]. Regardless of differences across the studies in conceptualisation of the MED (MDS or PREDIMED score, a posteriori-derived MED DP) and physical function outcomes, the similarity in the findings suggest that this DP may be ‘myoprotective’ in different populations of older adults. Therefore, the potential ‘myoprotective’ mechanisms that may underpin the effect of the MED on muscle are explored in Section 3. Conversely, the most consistent evidence obtained from a posteriori method implies that higher adherence to a westernized DP is a strong risk factor for impaired mobility and physical performance [95,99,113,116,120], which in turn might indicate lack of consumption of foods that characterise the MED pattern. Inconclusive results were observed with DPs and other components of sarcopenia, including muscle mass and muscle strength. More research and cross-validation of the finding are needed to understand the DP-sarcopenia relationship using well-defined cohorts of older adults with longer follow-up, which will allow for data harmonisation and pooled analyses of DP effects on muscle health and function and to define of an ‘optimal’ diet for prevention of sarcopenia. 

Based on the evidence reviewed here, the MED may have the best profile foods with ‘myoprotective’ properties and may provide a blueprint for designing DPs for muscle health in older adults in the future to include not only consideration of the type of classic MED foods but also their amount and ratio. Specifically, the traditional MED is characterised by higher intake of plant foods and olive oil (sources of vitamins, minerals, fibre, phytochemicals, healthy fatty acids, and protein), and moderate to low intake of animal-based foods, when compared with westernized DPs. The sources, amounts, and ratio of the profile foods in the MED may translate into favorable nutrient profiles for muscle health and function. However, there may be debate about how certain elements of the MED align with current nutritional guidelines (e.g., [132,133]) or expert opinion about nutrient needs for muscle health [44,52]. These may include a somewhat lower protein intake (when expressed as either percentage of total energy, or g/kg of body weight (actual or ideal) [134]) than recommended for older adults for muscle health [44,52] and lower vitamin D intake [discussed in 132]. Therefore, when designing an ‘optimal’ diet for prevention of sarcopenia around the MED, there may be other aspects of healthy diet, such as optimal protein intake for muscle health in older adults [44], that also need to be considered. 

## 3. Potential Mechanisms of Myoprotective Dietary Patterns in Older Adults 

In recent years, a number of systematic/narrative reviews and opinion articles have discussed the potential role of nutrition, nutritional supplements (e.g., protein supplements, vitamin D, n-3 fatty acids, creatine), and nutrients from foods (e.g., dairy protein and magnesium) in muscle ageing and sarcopenia e.g., [23,24,25,55,135,136,137,138,139,140,141,142,143,144,145,146,147,148]. Fewer reviews have examined the relationships between whole foods (e.g., fruits and vegetables, dairy, meats) [149,150,151,152,153], DPs (e.g., the MED; diet quality defined by dietary indices), and muscle health in older adults [56,57,58,59]. Many have hypothesised potential mechanisms by which nutrients (supplemented or from diet) may influence sarcopenia and age-related functional decline while also recognising the gaps in understanding based on current knowledge. For example, in a review about nutritional influences on muscle ageing, Welch [138] evaluated established, less established, and potential nutritional factors implicated in the main biomechanisms of age-related muscle loss. Whilst protein, essential amino acids (EAA), and leucine (mostly in combination with exercise) have been recognised as established factors, current understanding about the role of vitamin D, antioxidant nutrients (vitamin C, E, carotenoids), minerals, and trace elements (e.g., selenium, zinc, potassium, magnesium, iron, phosphorus) in muscle loss is less established. Conversely, the associations between dietary acid-base load (alkaline diets), dietary fat (type, composition and ratio), and bioactive compounds (nitrate, curcumin, antioxidant compounds in olive oil) are the least established—though they are recognised as potential factors relevant for muscle ageing [138]. Other nutritional factors and novel dietary candidates for muscle health suggested to contribute to healthy muscle ageing include dairy bioactive components, ursolic acid, phytochemicals nitrate-rich foods, and amino acid metabolites and precursors [23,25,135,136]—acting via a number of mechanisms including anti-inflammatory, anti-oxidative, and anabolic-promoting functions.

While much of the epidemiological research has focused on dietary intakes, there is also evidence of differences in nutrient status related to muscle health. For example, nutrient intake and biochemical nutrient status for a number of nutrients differs between sarcopenic and non-sarcopenic older adults regardless of energy intake, suggesting the importance of diet quality and nutrient density in the whole diet/DP for muscle health [46,154]. In the Maastricht Sarcopenia Study of 227 older adults aged ≥65 years, those with sarcopenia had 10–18% lower intake of five nutrients (n-3 fatty acids, vitamin B_6_, folic acid, vitamin E, and magnesium), their biochemical markers for n-6 fatty acid (linoleic acid) were 7% lower, and their homocysteine levels were 27% higher compared with non-sarcopenic older adults. The groups did not differ in energy intake [154]. In the PROVIDE study, a multi-centre randomized controlled trial of vitamin D and leucine-enriched whey protein supplements for sarcopenia conducted with 380 older adults aged ≥65 years, those with sarcopenia had ~6% lower protein intake (g/kg body weight), ~33% lower vitamin D, ~22% low vitamin B_12_, and ~2–6% lower intake of magnesium, phosphorus, and selenium compared with those without sarcopenia. However, of all biochemical markers examined, only the serum concentration of vitamin B_12_ was ~15% lower in sarcopenic older adults. Again, energy intake was similar in both groups [46]. 

These findings (and the results from the studies of DPs) suggest that combined actions (probably synergistic, cumulative, and antagonistic) of a number of macro- and micro-nutrients, together with other food components within the food matrix rather than a single nutrient, may be important for muscle mass, strength, and function in older adults. A higher intake of beneficial foods in a healthy diet may provide not only sufficient energy but also adequate levels of relevant ‘myoprotective’ nutrients and bioactive compounds per calorie. The interaction between the nutrients acting upon the aged muscle may help to preserve or improve myofibre quantity and quality by counteracting the pathophysiology of sarcopenia [34,36,37,38,39,123,127,128,130]. 

### Potential Myoprotective Mechanisms of Healthy Dietary Patterns: Example of Mediterranean Diet

**Figure 1:**
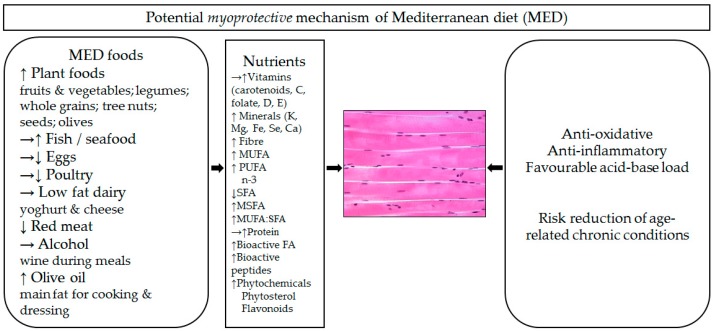
Hypothesised ‘myoprotective’ effect of the Mediterranean diet (MED). Because of a higher (↑) intake of plant-based foods, olive oil as a main source of fat, moderate-to-high (→↑) intake of fatty fish, moderate-to-low (→↓) intake of poultry and eggs, moderate (→) intake of dairy (mostly from yoghurt and cheese), low (↓) intake of red meats and meat products, and moderate intake of red wine during meals, the MED is a potential source of bioactive nutrients that may act synergistically, antagonistically, and cumulatively on the ageing muscle and may be ‘myoprotective’. Potential ‘myoprotective’ effects of the MED may work through its higher anti-oxidative and anti-inflammatory capacities, its favourable acid-base load (directly), and by its reducing of the risk of age-related conditions related to sarcopenia (indirectly). MUFA, monounsaturated fatty acids; PUFA, polyunsaturated fatty acids. Skeletal muscle image adapted from: https://teaching.ncl.ac.uk/bms.

The MED is recommended for healthy ageing and chronic disease risk reduction, not only because of higher intake of specific foods (e.g., plant-based foods, extra virgin olive oil) and nutrients (e.g., monounsaturated fatty acids (MUFAs) from olive oil and polyunsaturated fatty acids (PUFAs) from fish) but as a healthy eating pattern that as a whole exerts important health benefits [132], including those that may be ‘myoprotective’. Figure 1. depicts hypothesised ‘myoprotective’ effects of the complex combination of nutrients within a variety of foods in the MED on aged muscle, which may be indirect and direct. The indirect ‘myoprotective’ mechanisms of the MED may act through a reduction in risk of age-related chronic conditions (e.g., cardiovascular diseases, diabetes, polypharmacy) that are associated with either induction or worsening of sarcopenic symptomatology [155,156,157]. The MED may also act directly by ameliorating processes that have been implicated in sarcopenia (i.e., oxidative stress [127,128], inflammation [33,34,125,126,127], insulin resistance [129,130,131], and metabolic acidosis reviewed in [23,24,138,158,159] (Figure 1). 

Oxidative stress or accumulation of reactive oxygen and nitrogen species (ROS/NRS) in aged muscle leads to impaired cellular homeostasis and damage to key cell macromolecules such as proteins, lipids, and nucleic acid, affecting their structure and function reviewed in [127,128]. An excess of free radicals is pathologic and the inability of endogenous antioxidant enzymes to neutralise their detrimental effects on organelles and biomolecules in myofibres has been implicated in the loss of muscle mass, quality, and function [127,128]. A diet rich in exogenous antioxidants vitamins (e.g., vitamin E, C, carotenoids), minerals (zinc, coper, iron, selenium, magnesium) [148], and phytochemicals [160] from fruits, vegetables, olive oil, and nuts such as the MED can help in restoring the redox homeostasis [161] in the muscle and counteract ROS/NRS-induced damage. Antioxidants act as scavengers of excess free radicals produced by several organelles in myofibres and help maintain neuromuscular integrity and function that is changed with ageing [162]. However, because older myofibres benefit from the production of low-dose ROS to improve endogenous antioxidant system and cellular repair capacity during, for example, non-exhaustive exercise and lifelong physical activity, excess intake of antioxidant supplementation may be counteractive and cause reductive stress, as reviewed in [25,163,164]. Conversely, the MED may provide the right combination of antioxidants in the amounts beneficial to redox homeostasis in physically active older adults, thus improving function. 

In addition to modulating redox balance, antioxidants in the MED may affect signaling pathways and transcriptional factors such as nuclear factor κB (NK-κB) [165], which are responsible for the expression of pro-inflammatory cytokines, including interleukin 6 and 8 (IL-6 and IL-8) [165] and tumor necrosis factor α (TNF-α) [166]. Ageing is associated with a two-to-four-fold increase in pro-inflammatory mediators in serum or plasma, which can both induce and exacerbate age-related pathologies [165], including loss of muscle mass and function [33,34,125,126,127]. Besides chronic disorders and subclinical infections, several factors have been recognised to contribute to chronic low-grade inflammation (inflammageing), including increases in adipose tissue, hormonal changes, dysregulation of the immune system at the organ level [166], oxidative stress, mitochondrial dysfunction, glycation, telomere attrition, cell senescence, and epigenetic modifications at the cellular level [167]. Furthermore, inflammatory mediators (e.g., IL-6 and TNF-α) interfere with anabolic signaling by, for example, downregulating insulin and insulin-like growth factor-1 (IGF-1) and affecting muscle protein synthesis (MPS) after a meal or exercise [168,169]. Muscle insulin resistance may also arise in the presence of a high fatty acid availability and fat accumulation within the fibres with the induction of intracellular inflammation [170]. 

It has been proposed that inflammatory markers may act not only as common risk factors for the most age-related chronic conditions but also provide a link between lifestyle factors, chronic diseases (including sarcopenia), and physiological changes with ageing [166]. Several dietary components in a healthy DP may have anti-inflammatory properties [170], and the lack of them may negatively influence muscle ageing. Anti-inflammatory effects of the MED for muscle health may be achieved through higher intake of n-3 PUFA (fatty fish) and MUFA (olive oil). For example, a positive association between fatty fish intake and grip strength has been reported in older adults from the Hertfordshire Cohort Study—each additional portion of fatty fish was associated with a 0.43 kg increase in grip strength in men and a 0.48 kg increase in grip strength women [113]. In a 24-week intervention study involving 63 women (aged 65–70), a healthy diet rich in n-3 PUFAs (achieved through higher fish/seafood intake of ≥500 g/week) in combination with resistance exercise (RE), resulted in a 23% increase in type IIA muscle fibres and a downregulation of genes involved in inflammation and upregulation of regulators of growth response in the muscle [171], when compared with RE alone. Combining RE with a diet characterised by low n-6 to n-3 PUFA ratio (<2) and keeping the total fat energy intake mainly from MUFA and PUFA (and accord with the MED) may counteract age-related changes in fast myofibres. A traditional MED provides about 36–40% of total energy intake from fat (7–10% from saturated fats, 19–25% from MUFA, and 3–6% from PUFA) [133], because of a higher consumption of olive oil and a moderate to lower intake of dairy and meats compared to Western DPs. The beneficial effects of MED-like DPs on muscle function and physical performance compared to westernized DPs (e.g., [84,86,87,89,91,93,94,99]) may be attributed to the anti-inflammatory properties of its fat content and composition [160]. 

Better balance in the dietary acid-base load and potential reduction of diet-induced metabolic acidosis is another beneficial element of the MED ‘myoprotective’ mechanism that may be involved in muscle ageing and sarcopenia. Metabolic acidosis is a major cause of muscle loss in patients with chronic kidney disease, and the MED has been recently recommended in clinical guidelines as a strategy for chronic kidney disease prevention [172]. Because the MED is more plant-based, higher in fibre and lower in meats and dairy, it has potential to be less acidic. Though limited, research has shown that alkaline diets have positive effects on lean muscle mass in healthy older adults (reviewed in [23,24,138,158,159,173]). 

In conclusion, the MED may be beneficial for muscle health by simultaneously affecting several processes involved in sarcopenia and other age-related diseases not limited to oxidative stress and inflammation (Figure 1). 

## 4. Challenges Facing Dietary Patterns Research in Nutritional Epidemiology of Muscle Ageing

**Figure 2:**
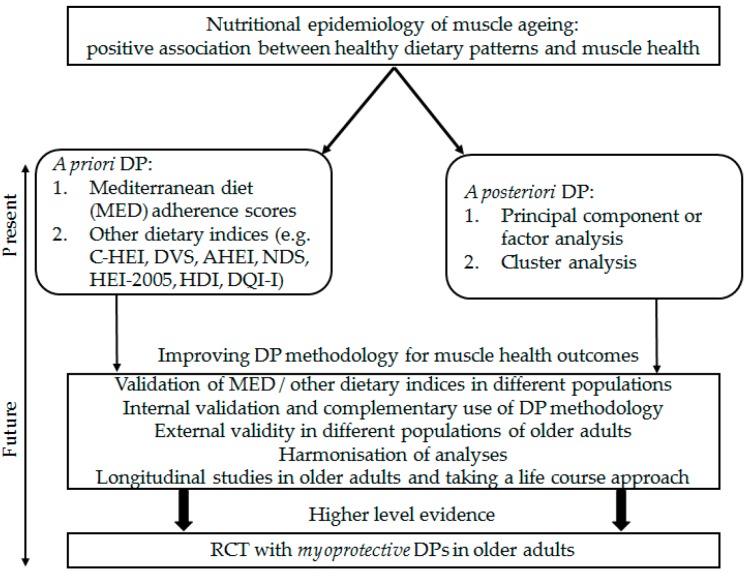
Dietary pattern-muscle health hypothesis investigation in nutritional epidemiology of muscle ageing faces several challenges to reach a higher level evidence. AHEI, Alternative Healthy Eating Index; DVS, Dietary Variety Score; DP, dietary pattern; HDI, Healthy Diet Indicator; HEI-2005, Healthy Eating Index 2005; DQI-I, Diet Quality Index-International; MED, Mediterranean diet; NDS, Nordic Diet Score.

Similar to research involving other health outcomes (e.g., cognition [174]), testing the diet-muscle health hypothesis using DP methodology faces several challenges. Figure 2 describes current approaches in nutritional epidemiology of muscle ageing and suggestions for improving DP methodology aimed to achieve a higher level of evidence in the future. For example, regardless of positive associations between the MED and physical performance in older adults from the Mediterranean and non-Mediterranean regions reported in several studies, different MED indices have to be cross-validated and should be based on the latest scientific understanding of the MED [81]. DP methodology is flexible and complementary techniques have been developed [67,68,69], but not all of them have been employed to test DP-muscle ageing hypothesis. Specifically, the RRR method not yet used in the studies of muscle ageing will allow investigation of DPs that maximally explain the variation in other response variables relevant for muscle health (e.g., protein and beneficial fatty acids) that are hypothesised to be associated with aetiology of sarcopenia and will provide mechanistic insights [69]. Harmonising the operational definitions of sarcopenia [26,106,172,175] and understanding of the concepts of ‘healthy’ diets for muscle health across the well-conducted prospective studies with longer follow-up will build on current scientific knowledge about the role of whole diet in muscle ageing. Though the research focus of most observational studies on diet and sarcopenia has been later life, there is evidence for benefits of a life course approach when investigating factors affecting sarcopenia. This includes the role of early nutrition in determining peak muscle strength in mid-life and consequent rate of muscle mass/strength decline in later life [176,177]. 

The proposed ‘myoprotective’ potential of a healthy DP such as the MED may be viewed as a sum of the different effects of multiple, distinct components, which do not act in isolation [178] and may vary across the regions (e.g., Mediterranean and non-Mediterranean regions) and over time [76,81]. Different compounds in the whole diet associated with better muscle health may have multifactorial effects through different mechanisms of action (including effects on single, multiple, or more general pathways), which may be also influenced by heterogeneous nature of sarcopenic muscle in older adults. 

In summary, to reach a higher-level evidence, harmonisation, validation (internal and external) of DPs, and complementary use of DP methodology in longitudinal/cohort studies of ageing with longer follow-up taking a life course approach in different populations is needed to gather information that will aid design of future randomised controlled trials (RCTs) with ‘myoprotective’ diets (Figure 2). 

## 5. Conclusions 

Muscle health is an integral part of healthy ageing. In recent years, diet/nutrition as a modifiable risk factor has been extensively investigated in relation to several aspects of muscle health, including muscle mass, strength, and physical performance. The evidence from nutritional epidemiology to date suggests a positive association between healthier DPs such as the MED and muscle function, including physical performance and mobility decline in older adults. The MED may exert ‘myoprotective’ effects through different mechanisms of action arising from multiple dietary components and pathways. Though there are few studies of sarcopenia, observational studies examining the role of whole diet are emerging and are showing a lower risk of sarcopenia in association with healthier diets. However, much of the current evidence is limited to cross-sectional studies and for some outcomes, such as muscle strength, and findings are mixed. To date, only one meta-analysis has been conducted that investigated the protective role of the MED in functional disability in older adults [64]. There are, therefore, clear gaps in knowledge. To reach a higher level of evidence, cross-validation and harmonisation of the methods to define dietary patterns are needed, together with studies of different populations of older adults with longer follow-up taking a life course approach, which may inform the design of future clinical trials. 

## Figures and Tables

**Table 1:** A priori dietary patterns and muscle health: Summary of evidence.

Ref.	Study Design/Duration	Participants/Setting	Measure of Muscle Health/Function	Dietary Assessment	Findings
**Summary of systematic reviews ^a^**					
[57] ^b^	1 longitudinal/4 years [83]; 1 cross-sectional [84];	older adults (aged ≥65), Hong Kong [83]; women (aged ≥50), the TwinUK registry [84];	prevalent and incident sarcopenia [83]; muscle mass and leg explosive power [84]	FFQ/MDS score [83,84];	→association between MDS and sarcopenia [83]; ↑association between %FFM and higher MDS, ↑leg extension power with ↑MDS [84]; Systematic review conclusion: Inconclusive evidence for the role of the MED in musculoskeletal health across the life course;
[59] ^b^	1 mixed (longitudinal and cross-sectional)/3 years [85]; 2 longitudinal [86,87]/9 and 8 years, respectively; 5 cross-sectional [88,89,90,91,92]^c^;	women (aged 65–72), Kupio, Finland [85]; older adults (aged ≥65), the InCHIANTI Study, Italy [86]; older adults (aged 70-79), the Health ABC Study, US [87];	10-m walking speed, chair rises, one leg stance, knee extension, grip strength, squat, LBMQ [85]; SPPB [86]; 20-m walking speed (usual and rapid) [87];	3-day food record/MDS [85]; FFQ/MDS score [86,87];	↑association between MDS walking speed, LBMQ and squat test, lowest MDS quartile associated with greater loss of relative skeletal muscle and lean mass, no other associations [85]; ↑association between MDS and SPPB at baseline, higher MDS associated with less decline in SPPB at 3-, 6-, 9-year follow-ups, higher MDS associated with lower risk of mobility disability, and slower decline in mobility [86]; higher MDS associated with higher usual and rapid walking speed and less decline in rapid walking speed [87]; Systematic review conclusion: Lower risk of sarcopenic symptomatology with higher adherence to the MED;
[58] ^b^	2 longitudinal [93,94]/3 and 2 years, respectively;	older adults (aged ≥60), the Senior-ENRICA, Madrid, Spain [93]; older adults (aged ≥65), the Three-City-Bordeaux Study, France [94];	physical function limitations (Rosow and Breslau scale, SF-12) [93]; modified frailty phenotype (Rosow and Breslau scale, chair stands) [94];	diet history/MDS, MEDAS [93]; FFQ/MDS [94];	→association between MDS, mobility impairment, and agility (Rosow and Breslau scale), →association between MDS and physical function decline (SF-12), the highest tertile of MEDAS associated with the decreased risk of developing agility, mobility, and physical functioning impairments [93]; the highest MDS associated with the reduced risk of developing mobility and lower extremity impairments [94]; Systematic review conclusion: Higher adherence to the MED associated with the lower risk of frailty and functional impairment, →association between the MED and sarcopenia in cohort studies, ↑association between the MED and sarcopenia in cross-sectional studies;
[56] ^b,d^	2 longitudinal [73,95] ^b^/6 and 3.5 years, respectively ^b^; 2 cross-sectional [96,97] ^d^; 2 longitudinal [83,98] ^d^/4 and 3 years, respectively;	older adults (aged ≥65), the InCHIANTI, Italy [73]; older adults (aged ≥60), the Senior-ENRICA, Madrid, Spain [95]; older adults (aged ≥60), NHANES 1999–2002, US [96]; older adults (aged ≥60), the Falls Risk and Osteoporosis Longitudinal Study, Australia [97]; older adults (aged ≥65), Hong Kong [83]; older adults (mean age 74.6 years), NuAge cohort, Quibeck, Canada [98];	grip strength, walking speed (over 15 feet) [73]; grip strength, 3-m walking speed [95]; knee extensor power, 20-feet gait speed [96]; lean mass, SPPB [97]; prevalent and incident sarcopenia [83]; grip strength, knee extensor, and elbow flexor strength [98];	FFQ/MDS score [73]; diet history/MDS, MEDAS score [95]; 24-hr multi-pass dietary recall/HEI-2005 [96]; FFQ/HEI, HDI [97]; FFQ/DQ-I [83]; three 24-hr dietary recalls/C-HEI [98];	higher MDS associated with the reduced risk of developing low walking speed, →association between MDS and grip strength [73]; →association between MDS, walking speed and grip strength, but the highest MEDAS tertile associated with reduced risk of low walking speed [95]; ↑association between HEI-2005, knee extension power and gait speed, but attenuated by physical activity [96]; →association between lean mass, SPPB, and HEI, ↑weak association between lean mass and HDI (women), and SPPB and HDI (men) [97]; the highest quintile of DQ-I associated with the reduced risk of prevalent sarcopenia (men), →association between DQ-I and 4-year incident sarcopenia (men and women) [83]; →association between C-HEI and muscle strength (grip strength, knee extensor, elbow flexor) maintenance over 3 years [98]; Systematic review conclusion: Strong observational evidence for the association between ‘heathier’ diets and lower risk of decline in physical performance, but not for decline in muscle strength;
**Additional evidence ^e^**					
[99] ^b,d^	cross-sectional;	older adults (aged 55–80 (men), and 60–80 (women)), Balearic Islands and Madrid, Spain;	physical fitness (grip strength, 30-s arm curls, 30-s chair stand, 8-foot TUG, 30-m gait speed, 6-min walk test);	FFQ/MED and Westernized DP/factor analysis;	↑association between the highest quartile of the MED and 30-s chair stands (men), and 6-min walking speed (women), →association between the MED and muscle strength (grip strength and arm curls) in both sexes, the highest MED quartile associated with less time to complete TUG test and 30-m gait speed (men), higher quartiles of a ‘Westernized’ DP associated with slower gait speed, lower body strength (chair rises), agility (8-foot TUG), and aerobic endurance (6-min walk test) in both sexes;
[100] ^b^	cross-sectional;	older women (aged 60–85), the PERSSILAA study, Campania Region, Italy;	grip strength;	7-day food records/PREDIMED score;	women with higher grip strength had higher PREDIMED scores and were more likely to belong to the ‘high adherence’ PREDIMED group (score ≥10);
[101] ^b,d^	longitudinal/10 years;	older adults (mean age 61.6 years), the Helsinki Birth Cohort Study, Finland;	mobility limitations;	FFQ/mMDS /NDS;	→association between mMDS and mobility limitations at 10-year follow-up, the highest tertile of NDS associated with the lower risk of developing mobility limitations;
[102] ^b^	cross-sectional;	type-2 diabetes patients (aged >60 years), the Center for Successful Aging, Diabetes at Sheba Medical Center, Israel;	Berg balance test, TUG, 6-min walk test, 10-m walk test, four square step test, 30-s chair stand, grip strength;	FFQ/MDS;	↑association between MDS and grip strength, but not after adjustment for key covariates, age × MDS interaction: The highest MDS tertile associated with longer distance achieved in 6-min walking test, faster time 10-m walk, and better balance score in those aged ≥75 years;
[103] ^b^	longitudinal/10 years;	older adults (aged >60), the Helsinki Birth Cohort Study, Finland;	grip strength, leg strength, lean body mass	FFQ/MDS;	↑association between grip strength and NDS (women) at 10-year follow-up, →associations between NDS and muscle mass in both sexes;
[104] ^d^	longitudinal/15 years;	older men (aged 66 at baseline)/the British Regional Heart Study, UK;	mobility limitations (going up or down stairs or walking 400 yards);	FFQ/HDI/EDI;	the highest HDI and EDI category at baseline associated with the reduced risk of mobility limitations 15 years later;
[105] ^d^	cross-sectional;	older adults (aged ≥65), the National Fitness Award project, the Ministry of Culture, Sports, and Tourism, South Korea;	fitness tests (2-min step test, TUG, figure-of-8 walk test, grip strength, arm curls);	FFQ/RFS;	↑association between RFS and grip strength (women), →association between RFS and other physical performance tests;
Evidence summary for studies with a priori DPs: Higher adherence to the MED associated with better lower extremity functioning, mobility, better walking speed, and less decline over time; inconclusive evidence for muscle strength; mixed evidence for Diet Quality Indices and sarcopenia/elements of sarcopenia; emerging evidence for ↑association between ‘healthier’ DP and mobility/mobility limitations.					

^a^ Summary of selected observational studies from the systematic reviews described in Section 2.1.2, Section 2.1.3 (Mediterranean diet), Section 2.2and Section 2.2.1 (Diet Quality Indices) (publication years 2017–2018; cut-off for inclusion April 2017). ^b^ Reviews and studies using Mediterranean diet indices only. ^c^ Not described in detail in this review. ^d^ Studies using Diet Quality Indices and region-specific a priori diet scores. ^e^ Cut-off for inclusion January 2019. ↑: Positive association; →: No association; C-HEI, Canadian-Healthy Eating Index; DQ-I, Diet Quality Index-International; DP, dietary pattern; EDI, Elderly Dietary Index; %FFM, percent fat free mass; FFQ, Food Frequency Questionnaire; HDI, Healthy Diet Indicator; HEI, Healthy Eating Index; HEI-2005, Healthy Eating Index-2005; Health ABC Study, the Health, Aging, and Body Composition Study; InCHIANTI Study, the Invecchiare in Chianti Study; LBMQ, lower body muscle quality; MDS, Mediterranean Diet Score; MED, Mediterranean diet; MEDAS, Mediterranean Diet Adherence Screener; NHANES 1999–2002, the National Health and Nutrition Examination Survey 1999–2002; NuAge Study, the Quebec Longitudinal Study on Nutrition and Aging; PERSSILAA, the PERsonalised ict Supported Services for Independent Living and Active Ageing; PREDIMED, the Prevención con Dieta Mediterránea; RFS, Recommended Food Score; SPPB, Short Physical Performance Battery; TUG, Timed Up-and-Go Test.

**Table 2:** A posteriori dietary patterns and muscle health: Summary of evidence.

Ref.	Study Design/Duration	Participants/Setting	Measure of Muscle Health/Function	Dietary Assessment	Findings
**Summary of systematic review ^a^**					
[56]	4 cross-sectional [111,112,113,115]; 3 longitudinal [114,116,117]/3.5 to 16 years;	older adults (aged ≥65), the KNHANES, South Korea [112]; older adults (aged 59–73), the Hertfordshire Cohort Study, UK [113]; older adults (mean age 68 years), the Hertfordshire Cohort Study, UK [115]; older adults (aged ≥55), Tehran, Iran [111]; older adults (aged ≥60 at the final follow-up) [114]; older adults (aged ≥85), the Newcastle 85+ Study, UK [116]; older adults (aged ≥60), the Senior-ENRICA, Madrid, Spain [117];	appendicular skeletal muscle mass [112]; grip strength [113]; short SPPB (3-m walk test, 5 chair rises, one-legged standing balance [115]; 8-feet walking speed [114]; sarcopenia [111]; grip strength, TUG [116]; grip strength, 3-m walking speed [117];	24-hr dietary recall/clustering analysis [112];FFQ/PCA [111,113,114,115]; 24-h dietary recall/cluster analysis [116]; diet history/PCA [117];	a ‘Westernized Korean’ DP associated with increased abnormalities in lean mass compared with a ‘Traditional Korean’ DP [112]; ↑association between ‘Prudent diet’ and grip strength, but partly explained by fatty fish consumption in men and not in women [113]; →association between diet and SPPB in men, higher ‘Prudent diet’ score associated with better 3-m walk time chair stands and balance in women, but not after adjustment for key covariates [115]; the highest tertile of a ‘MED-like’ DP associated with the reduced risk of sarcopenia, but →association between a ‘Westernized’ DP and sarcopenia [111]; →association between a ‘Healthy-foods’ DP and physical function, the highest tertile of a ‘Western-type’ DP associated with the increased risk of poor physical function [114]; men belonging to ‘High Red Meat’ DP had worse GS at baseline, and greater GS decline over 5 years [116]; →association between a ‘Prudent diet’ and grip strength, a ‘Westernized’ DP associated with slow walking speed [117]; Systematic review conclusion: The evidence for the association between ‘heathier’ diets, physical performance and muscle strength has been mixed;
**Additional evidence ^b^**					
[118]	longitudinal/3 years;	older adults (aged ≥85), the Newcastle 85+ Study, UK;	sarcopenia;	24-h dietary recall/cluster analysis;	‘Traditional British’ DP associated with the increased risk of sarcopenia at baseline and 3-year follow-up in older adults with good protein intake;
[119]	longitudinal/10 years;	older adults (aged ≥67), the Three-City Bordeaux Study, France;	mobility limitations (Rosow-Breslau scale);	FFQ/hybrid clustering method;	‘Biscuits and snacking’ cluster associated with a 3-fold increased risk of mobility restriction compared with a ‘healthy cluster’ in men; →association between clusters and mobility in women;
[120]	cross-sectional;	older adults (aged ≥60), the KNHNES 2008–2011, South Korea;	appendicular skeletal muscle mass;	FFQ/factor analysis;	a ‘Healthy’ DP associated with higher muscle mass in men, but not in women;
[121]	cross-sectional;	older adults (aged ≥70), Gipuzkoa, Spain;	TUG;	FFQ/multiple correspondence and cluster analysis;	three DPs with progressively worse adherence to dietary recommendations; a gradient effect of DPs in relation to TUG;
[122]	longitudinal/60–64 years;	British 1946 birth cohort, the MRC National Survey of Health and Development study, UK;	chair rises, TUG, and standing balance (at age 60–64);	prospective 5-day food diaries (completed in 1982, 1989, 1999, and 2006–2010)/PCA;	↑association between a ‘healthier’ DP at ages 36, 43, 53, and 60–64 and physical performance at age 60–64;
Evidence summary for studies with a posteriori DPs: Mixed evidence for ‘healthier’ DPs and components of sarcopenia; higher adherence to ‘Westernized’ DPs associated with impairments in mobility and physical performance.					

^a^ Summary of selected observational studies described in Section 2.2. ^b^ Summary of observational studies described in Section 2.3.1. ↑: Positive association; →: No association; DP, dietary pattern; FFQ, food frequency questionnaire; MRC, Medical Research Centre; PCA, principal component analysis; KNHANES, Korea National Health and Nutrition Examination Survey; TUG, Timed Up-and-Go Test.
